# Raccoon dog rabies surveillance and post-vaccination monitoring in Lithuania 2006 to 2010

**DOI:** 10.1186/1751-0147-53-58

**Published:** 2011-11-15

**Authors:** Dainius Zienius, Gediminas Pridotkas, Raimundas Lelesius, Vilimas Sereika

**Affiliations:** 1 Lithuanian University of Health Sciences, Veterinary Academy, Veterinary Institute, Tilzes 18, Kaunas, Lithuania

## Abstract

**Background:**

Oral rabies vaccination (ORV) in rabies infected regions should target the primary rabies vector species, which in Lithuania includes raccoon dogs as well as red foxes. Specific investigations on ORV in raccoon dogs are needed e.g. evaluation of vaccine effectiveness under field conditions. The objective of the current study was to investigate the efficacy of the ORV programme 2006-2010 in Lithuania by examining the number of rabies cases and estimating the prevalences of a tetracycline biomarker (TTC) and rabies virus antibodies in raccoon dogs.

**Methods:**

From 2006 to 2010, 12.5 million rabies vaccine-baits were distributed by aircraft. Baiting occurred twice per year (spring and autumn), targeting raccoon dogs and red foxes in a 63,000 km^2 ^area of Lithuania. The mandibles of raccoon dogs found dead or killed in the vaccination area were analyzed by fluorescence microscopy for the presence of the TTC. Rabies virus sera neutralizing anti-glycoprotein antibody titres were determined using an indirect ELISA method and seroconversion (> 0.5 EU/ml) rates were estimated.

**Results:**

During the study period, 51.5% of raccoon dog mandibles were positive for TTC. 1688 of 3260 tested adults and 69 of 175 tested cubs were TTC positive. Forty-seven percent of raccoon dog serum samples were positive for rabies virus antibodies. 302 of 621 investigated adults and 33 of 95 investigated cubs were seropositive. In the same time 302 of 684 and 43 of 124 tested samples were TTC and ELISA positive in spring; whereas 1455 of 2751 and 292 of 592 tested samples were TTC and ELISA positive in autumn. There was a positive correlation between the number of TTC and antibody positive animals for both adult and cub groups.

**Conclusions:**

ORV was effective in reducing the prevalence of rabies in the raccoon dog population in Lithuania. The prevalence of rabies cases in raccoon dogs in Lithuania decreased from 60.7% in 2006-2007 to 6.5% in 2009-2010.

## Background

The red fox (*Vulpes vulpes*) continues to be the principal vector and reservoir for sylvatic rabies in Europe, even though its role has been drastically reduced in Western Europe by means of oral rabies vaccination (ORV) [1]. At the same time, raccoon dog (*Nyctereutes procyonoides*) rabies has increased in North-eastern Europe and more than doubled in the Baltic countries. Both red fox and raccoon dog rabies cases accounted for an even level of 90 to 94% of wildlife rabies cases in the last decade [2]. Raccoon dogs were introduced as fur animals in western parts of Russia in 1929-1955 from where they spread quickly throughout Europe [3]. That had a direct influence on the rabies epidemiology in North-eastern Europe, especially in Lithuania where the number of rabies cases in raccoon dogs has been similar or even higher than in red foxes [4]. The involvement of two component vectors can substantially alter epidemiology of the infection and might affect transmission within and between species. Interspecies transmission is likely to occur because of strong ecological links between raccoon dogs and red foxes. Also, overlapping territories have been found and animals have been observed in each other's vicinity [5]. However, empirical [5,6], theoretical [7] and phylogenetic [8] evidences suggest that the contact rates between red foxes and raccoon dogs produce a single epizootic in both species in North-eastern Europe. Nevertheless, the overall aim remains the elimination of terrestrial rabies from the whole of the enlarging European Union, including the Baltic countries, and beyond them. However, financial concerns demand an optimal balance of cost and benefits. Consideration includes the growing presence and spread of raccoon dogs, a significant host of rabies virus and a species with a high reproductive potential [7].

Although classical rabies virus strains are host-species specific they can be successfully controlled by ORV. This has been demonstrated in experimental and field studies, as well as during the ORV campaigns in Poland [9,2], Estonia [10,11] and Latvia [12]. Researches addressing the control of sylvatic rabies have focused on the development of vaccine and effective methods of ORV of wild vector species. Most oral rabies vaccines presently used in Europe contain modified-live attenuated virus originating from the Street Alabama Dufferin (SAD) Rabies Virus [13]. The different "SAD" oral vaccines were used in Europe to prevent rabies in red fox populations, but ORV programmes in rabies-infected countries should target not only red fox, but also the raccoon dog. Specific investigations concerning the ORV in raccoon dogs are needed, especially in Lithuania where rabies cases in raccoon dogs were higher than in red foxes. The best approach for testing a vaccine is to evaluate its effectiveness under field conditions. The objective of the current study was to investigate the efficacy of the ORV programme in Lithuania from 2006 to 2010 by examining the number of rabies cases and the prevalences of a tetracycline biomarker (TTC) and rabies virus antibodies in raccoon dogs.

## Methods

### Study area

During the time of investigation the vaccination area involved of the entire Lithuanian territory (63,000 km^2^). Lithuania is situated along the south-eastern shore of the Baltic Sea, it shares borders with Latvia (588 km) to the north, Belarus (677 km) to the southeast, Poland (104 km), and the Russian exclave of Kaliningrad (255 km) to the southwest. The vaccination area was chosen because of the natural barrier of the Baltic Sea to the west and typical raccoon dog/red fox areas of habitation including forests (35% of the territory), village surroundings and isolated bunch of trees, etc. combined with a high incidence of rabies cases. According to the hunting statistics, the total population of raccoon dogs was up to 40,000 with a density ranging between 0.3 and 1.45 animals/km^2^. The total human population is around 3 millions with a density of 22.7-50.5 persons/km^2 ^within the suburban and rural vaccination area including five major cities with a population over 100,000 citizens.

### Vaccine

Lysvulpen vaccine (Bioveta^®^, Czech Republic) was used for ORV campaigns. It contains a modified attenuated SAD Bern strain obtained from first attenuated ERA vaccine after propagation on canine and bovine kidney cells (biological activity 1.8 × 10^6^-1.8 × 10^8 ^TCID50/bait (Tissue Culture Infective Dose). Baits contain 1.8 ml of vaccine in a blister plastic capsule sealed with an aluminium foil that is embedded in the casing that contains 150 mg tetracycline hydrochloride (TTC) as a vaccination indicator (bio marker). Baits were stored at -20°C prior to use and during the vaccination period while vaccines were stored in refrigerated lorries at -20°C (monitored) during the entire campaign.

### Bait distribution

ORV in Lithuanian red foxes and raccoon dogs was organized according to the Lithuanian National Rabies Prevention Programme and implemented in 2006. The 2006 spring (March-May) ORV campaign was organized over 40,000 km^2 ^area in the south-eastern and central parts of Lithuania and 800,000 baits distributed. The 2006 autumn (October-December) vaccination covered the entire Lithuanian territory and 1.3 million baits were used. From 2007 to 2010 the ORV area was 63,000 km^2 ^in both spring and autumn - 1.3 million baits per campaign were used (i.e. 2.6 million vaccine baits per year). The baiting strategy was designed according to the epidemiological situation of rabies and investigations of wildlife populations and hunting statistics. Baits were distributed by four Cesna-type small airplanes at a density of approximately 20 baits/km^2^. The aircraft flying lines were separated by 1000 m; the flight altitude was lower than 250 meters and the speed 200-250 km/h with clear ground visibility. Flight lines and bait droppings were registered on the map using Geographical Positioning System (GPS) and data were recorded on Geographical Identification System (GIS).

### Sample collection

During the entire study period, all rabies-positive hunted, road-killed and otherwise dead animals from vaccination territories were included in the epidemiological investigation. A positive diagnosis was based on laboratory examination. Brain samples were collected on opening the skull in a necropsy room or by using the retro-orbital route for brain sampling [14]. Field blood samples were generally collected from the thoracic cavity of freshly killed raccoon dog. Samples were stored at 4°C for 24 hours to separate serum from clotted blood and serum was then stored at -20°C until use for ELISA testing. The raccoon dog lower jaws were collected at necropsy and canine teethes with surrounding alveolar bone tissues were isolated from each case.

### ORV programme monitoring

The ORV programme monitoring was based on analyzing the incidence of rabies in wildlife before and during ORV, testing of the occurrence of TTC and examining serum samples of the target animals for serological evidence of rabies [15]. Information about rabies occurrence in the raccoon dog population in Lithuania 2001-2010 was based on the data published in Rabies Bulletin Europe. The information in regard to biomarkers and serology investigations were based on the annual data summaries of the Lithuanian National Food and Veterinary Risk Assessment Institute (NFVRAI). Rabies diagnostic techniques have been standardized internationally [16,17] and immunofluorescent antibody test (IFAT) was used for the detection of rabies virus antigen. Bait uptake in raccoon dog was determined by detection of TTC lines in teeth and alveolar bone. One hundred μm sections of mandibular bone were analyzed without mounting medium by ultra-violet fluorescence microscopy (excitation filter 380-425 nm, barrier 460 nm) for the presence of specific green fluorescence. Serological response was determined using the indirect ELISA method (Bio-Rad Platelia Rabies II Kit, France). Assays were done in a 96 wells microplate, coated with rabies virus glycoprotein as previously described [18]. Antibody titres were expressed as equivalent units per ml (EU/ml). Seroconversion (> 0.5 EU/ml) rates were estimated [19].

### Statistical analyses

The statistical comparison in positive/negative samples of "Lysvulpen" bait uptake (TTC) and seroconversion in raccoon dog adults and cubs during the spring and autumn campaigns were done with calculation Chi^2^-squared (χ^2 ^test), *P - *probability and Fisher's exact - statistical significance tests [20-22].

## Results

**Figure 1:**
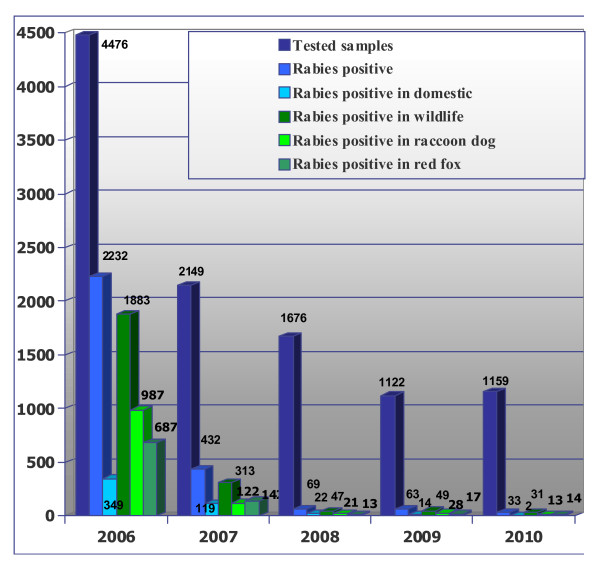
**Investigation of rabies epidemiological situation (cases per year) in Lithuania 2006 to 2010**.

ORV efficacy evaluation in the Lithuanian raccoon dog population was based on rabies surveillance before and during the prevention programme. In 2001-2006, 9,401 brain samples of rabies suspected cases were examined in Lithuania and rabies was confirmed in 76.1% (22.2% in domestic animals and 77.8% in wildlife). 36.3% of the positive wildlife cases were raccoon dogs and 31.6% were red foxes (details are provided in additional file 1: Examination of suspected rabies cases in Lithuania 2001-2006). In 2006-2010, a total of 10,582 brain samples were examined. The prevalences of positive cases in wildlife and domestic animals were 22.1% and 4.8%, respectively (Figure 1). From 2006 to 2008, the prevalence of rabies cases in raccoon dogs and red foxes decreased from 22.1% to 1.3% and from 15.3% to 0.8%, respectively. The same situation was observed in 2010, when among 1,159 rabies suspected samples tested, 13 positive raccoon dogs (1.1%) and 14 positive red foxes (1.2%) were found. During the vaccination period the prevalence of rabies infected raccoon dog decreased from 22.1% in 2006 to 1.1% in 2010. In 2006 and 2007, a total of 60.7% of raccoon dog brain samples were found rabies virus positive, whereas in 2009 and 2010 totally 6.5% rabies virus positive samples were detected in raccoon dogs. The hunting statistics in Lithuanian wildlife 2004-2010 shows that 3,439 raccoon dogs were killed in Lithuania in 2004, 2,818 in 2006, 5,554 in 2008 and 10,290 in 2010.

Between 2006 and 2010, 3,435 mandible specimens were tested for the TTC biomarker of which 1,757 (51.5%) were positive. The proportion of TTC positive adults was significant higher than for cubs (chi^2 ^= 10.14, *P *= 0.001) (Table 1). The stratified analysis revealed a significant difference between years for TTC results according to age of animals (Mantel-Haenszel chi^2 ^= 10.94; *P *= 0.0009). Annual data indicated that adults were more frequently marked than cubs, although data were only significantly different in 2006. When analysing data per campaign (Table 2), TTC marking was found more frequent after autumn campaigns than after spring campaigns (chi^2 ^= 16.7; *P *= 0.00004). The stratified analysis indicated a significant difference between years on TTC results according to the baiting time (Mantel-Haenszel chi^2 ^= 25.3; *P *= 5 × 10^-7^) as autumn campaigns induce a better marking than spring campaigns. However, the difference between baiting times were only significant for 2008 and 2010.

**Table 1:** Comparison of "Lysvulpen" bait uptake (TTC) in adults and cubs of raccoon dogs in the 2006 to 2010 oral rabies vaccination campaign in Lithuania

Year	Raccoon dog mandible samples									
	
	Adults TTC negative		Adults TTC positive		Cubs TTC negative		Cubs TTC positive		Chi^2^*	*P*
			
	n*	%	n*	%	n*	%	n*	%		
**2006**	720	58	304	42	53	75	13	25	6.49	0.01

**2007**	192	19	156	81	15	33	10	67	Fisher exact*	0.15

**2008**	1323	55	597	45	58	64	21	36	1.79	0.18

**2009**	331	42	192	58	6	33	4	67	Fisher exact*	0.5

**2010**	694	37	439	63	43	51	21	49	3.58	0.06

**Total**	3260	48	1688	52	175	61	69	39	10.14	0.001

Stratification: Chi^2 ^Mantel-Haenszel = 10.94; *p *= 0.0009

Chi^2 ^- squared test (χ^2 ^test)

*P - *probability

Fisher's exact - statistical significance test

n - number of samples

**Table 2:** "Lysvulpen" bait uptake (TTC) in Lithuanian raccoon dogs in the 2006 to 2010 oral rabies vaccination campaign during the spring and autumn campaigns

Year	Raccoon dog mandible samples									
	
	Spring TTC negative		Spring TTC positive		Autumn TTC negative		Autumn TTC positive		**Chi**^2^*	P*
		
	n*	%	n*	%	n*	%	n*	%		
**2006**	43	60	17	40	730	59	300	41	0.04	0.83

**2007**	38	24	29	76	169	19	137	81	0.44	0.5

**2008**	358	61	139	39	1023	53	479	47	6.85	0.009

**2009**	59	39	36	61	278	42	160	58	0.24	0.62

**2010**	186	56	81	44	551	31	379	69	37.7	0

**Total**	684	56	302	44	2751	47	1455	53	16.7	0.00004

Stratification: Chi^2 ^Mantel-Haenszel = 25.3; p = 5 × 10^-7^

Chi^2 ^- squared test (χ^2 ^test)

*P - *probability

n - number of samples

During the investigation, 716 serum samples were tested for rabies virus antibodies by ELISA, 335 were positive (> 0.5 EU/ml), i.e. 46.8%. Post mortem blood samples were more or less haemolysed (42% of samples showed severe haemolysis, 38% were slightly haemolysed and 20% had no signs of haemolysis). No significant differences in titres were observed in the geometric means. Analysis of serology data (Table 3) showed that generally the proportion of positive adults was significantly higher than for cubs (chi^2 ^= 6.39, *P *= 0.01). The stratified analysis revealed a significant difference between years on ELISA results according to age of animals (Mantel-Haenszel chi^2 ^= 5.01; *P *= 0.02). When considering serological results according to campaign (Table 4), the analysis showed that serological response were better after autumn campaigns than after the spring campaigns, i.e. titres were higher (chi^2 ^= 8.82; *P *= 0.003). The stratified analysis revealed a significant difference between years on ELISA results according to the baiting period (Mantel-Haenszel chi^2 ^= 6.38; *P *= 0.01).

**Table 3:** "Lysvulpen" seroconversion (analyzed by ELISA) in adults and cubs of raccoon dogs in the 2006 to 2010 oral rabies vaccination campaign in Lithuania

Year	Raccoon dog serum samples									
	
	Adults ELISA negative		Adults ELISA positive		Cubs ELISA negative		Cubs ELISA positive		Chi^2^*	P*
			
	n*	%	n*	%	n*	%	n*	%		
**2006**	79	63	29	37	18	89	2	11	4.4	0.03

**2007**	199	40	120	60	31	71	9	29	10.6	0.001

**2008**	151	46	82	54	18	39	11	61	0.3	0.58

**2009**	49	61	19	39	4	50	2	50	Fisher exact*	0.52

**2010**	13	64	52	36	24	63	9	38	0.01	0.91

**Total**	621	51	302	49	95	65	33	35	6.39	0.01

Stratification, Chi^**2 **^Mantel-Haenszel = 10.94; *p *= 0.0009

Chi^2 ^- squared test (χ^2 ^test)

*P - *probability

Fisher's exact - statistical significance test

n - number of samples

**Table 4:** "Lysvulpen" seroconversion (analysed by ELISA) in Lithuanian raccoon dogs in the 2006 to 2010 oral rabies vaccination campaign during the spring and autumn campaigns

Year	Raccoon dog serum samples									
	
	Spring ELISA negative		Spring ELISA positive		Autumn ELISA negative		Autumn ELISA positive		Chi^2^*	P*
			
	n*	%	n*	%	n*	%	n*	%		
**2006**	16	81	3	19	81	65	28	35	1.52	0.21

**2007**	30	63	11	37	200	41	118	59	5.26	0.02

**2008**	28	64	10	36	141	41	83	59	5.03	0.02

**2009**	11	36	7	64	42	67	14	33	Fisher exact*	0.07

**2010**	39	69	12	31	128	62	49	38	0.72	0.39

**Total**	124	65	43	35	592	51	292	49	8.82	0.003

Stratification: chi^2 ^Mantel-Haenszel = 6.38; *p *= 0.01

Chi^2 ^- squared test (χ^2 ^test)

*P - *probability

Fisher's exact - statistical significance test

n - number of samples

## Discussion

The use of attenuated rabies virus strains in vaccines has led to the elimination of sylvatic rabies from large areas of Europe. For 25 years, the basic concept of ORV focused on the red fox being the main rabies reservoir, but during the last 5 years raccoon dog mediated rabies expanded, mainly in the Baltic region and in Central Europe. This had a direct influence on the vaccination strategy. Fortunately, raccoon dogs respond well to ORV and all available vaccines currently used are efficient in both the red fox and raccoon dog [3,11,23]. Despite that the majority of live attenuated or sub-unit rabies vaccines are derived from viruses obtained 50-100 years ago [24], vaccine failures are rare. However, under specific circumstances they do occur and differences between "street" and vaccine ("fixed") strains may contribute to these failures [25]. The rabies epidemiological situation before the ORV programme implementation in Lithuania showed a rapid increasing number of rabies cases in raccoon dogs. Between 2001 and 2005, the average annual number of rabies cases in raccoon dogs was 322.8. In 2005 and in 2006 this number increased dramatically to 599 in 2005 and 987 in 2006. The 5 years of ORV with "Lysvulpen" (SAD Bern) vaccine was effective in reducing the prevalence of rabies cases in raccoon dogs in Lithuania. During the vaccination period (2006-2010), the number of raccoon dog rabies cases dropped from 987 in 2006 to 13 in 2010. However the investigation of vaccine baits acceptance evaluated by the presence of the TTC biomarker and antibody titres in raccoon dogs in a vaccination area were more informative. The TTC investigation in spring and autumn periods in 2006-2010 ORV indicated that 46.1% of adults and 17.1% of cubs examined in spring were positive, but in autumn campaigns the situation was different as 47.6% of cubs and 53.1% of adults were TTC positive. This means that 20-50% of tested target animals have had an oral contact with the vaccine baits and, possibly, were vaccinated. The hunting statistics indirectly showed that the raccoon dog population was increasing rapidly and a low prevalence of TTC biomarker positive animals might reflect of high population density and greater competition for baits from other target wildlife species, e.g. red foxes in urban and suburban areas.

Interpretation of TTC data in post mortem bone samples is difficult because this calciphilic marker is deposited in bones and teeth with a very slow turnover. As it was used extensively during the whole vaccination programme, its presence cannot always be reliably related to the time of consumption or a specific campaign. TTC data can only be correlated with findings in an area if not used within three years before ORV [26]. The TTC concentration in the vaccine baits was 150 mg (including the "Lysvulpen"). Prevaccination analysis of the baits showed that TTC was present in a 1:2 ratio between epitetracycline and tetracycline. If a vaccine bait was eaten by an animal shortly after distribution, it contained the marking potential equivalent to 91 mg TTC. If a bait remains in the environment for several days, the marking potential decreases by 40% [27]. Other limitations associated with the use of TTC as a biomarker for ORV is its low rate of incorporation in bones of adult animals (i.e. low growth rate of bones and teeth) [28].

Titration of rabies virus antibodies is a more specific test than analysing the TTC biomarker when evaluating of the efficacy of ORV campaigns. During spring and autumn vaccination campaigns, 37.1% and 48.8% of tested raccoon dog serum samples were ELISA positive, respectively. Similar 38.3% of adults and 18.2% of cups had ELISA positive serum samples during the spring while 52.2% adults and 28.8% cubs were seropositive during the autumn campaigns. The prevalences for the TTC bio marker and seropositivity were similar in cups during the spring vaccinations (17.0% and 18.2%, respectively), but rather different in autumn (47.6% TTC and 28.8% seropositive).

Totally 133 serum samples (105 of 621 tested adults and 28 of 95 of tested cubs) were negative for antibodies but positive for the TTC marker while the opposite situation was not observed. An ELISA test was developed for testing even highly haemolysed samples, because the fluorescent antibody virus neutralization test, like other cell culture based techniques, is too difficult for large scale screening and too sensitive to the cytotoxicity associated with poor quality samples [29,30]. The Platelia Rabies II kit was validated recently and performed well, i.e. on specificity, sensitivity, and repeatability [18,31]. It was found to be highly specific in all species (more than 98%) using a cut-off value of 0.5 EU/ml [28], which simplifies the interpretation of the results generated by the kit. The index of sensitivity was between 92.4% and 94.5% for fox samples, and reached 83% for domestic carnivores [18]. The analysis of 5 year ORV programme in Lithuanian raccoon dogs indicates that 16.9% of adults and 27.9% of cubs tested positive for the TTC biomarker had low or undetectable levels of rabies virus antibodies (< 0.5 EU/ml). The 92-96% seroconversion rates observed in the laboratory [3] were not reflected in the field trials.

Despite relatively low prevalences of antibody and biomarker positive raccoon dogs, we noted a significant reduction in rabies cases throughout Lithuania since the first ORV campaign in 2006. Protection against rabies despite the absence of detectable virus specific antibodies in serum is a classical result for the recombinant rabies vaccine [32], but this has also been described following administration of the SAG-2 oral vaccine [33,34]. In addition, low rate of seroconversion and low level of rabies virus neutralizing antibodies after oral vaccination, despite a high rate of protection after challenge, seem to be a common feature of the canine species [35]. During the entire ORV period, baits acceptance was significantly higher in October-November than in March-May. High differences in seroconversion rates were observed between populations of young and adult raccoon dogs comparing spring and autumn ORV campaigns. During the late spring and summer periods, only 9.2-18.3% of the juvenile raccoon dogs were protected by means of rabies antibodies. All these animals were TTC positive. As with red foxes [36], large numbers of young raccoon dogs may be unprotected against rabies infection during summer and early autumn seasons as cubs are too young to eat baits and their maternal antibodies can prevent active immunization during the first two months of life [3,37]. In Europe, red fox cubs are born from around 15 March to 15 April. Raccoon dogs have a longer gestation period than the red fox and most gives birth in May [38]. Theoretically, this effect could be minimized by delaying distribution of vaccine baits to later in the year (July) when cups become juvenile and thus eat baits and have lost maternal immunity. However, distribution of baits during the summer in Lithuania is problematic as "Lysvulpen" (SADBern) vaccine baits should not be exposed to temperature higher than 15°C according to the producer specifications.

## Conclusions

The positive results obtained in Lithuania demonstrated that the 2006-2010 wildlife ORV campaign was able to decrease the incidence of infection in the raccoon dog population. The investigation of TTC markers and serology indicated low number of vaccinated raccoon dogs in the juvenile subpopulation after the March-May period of ORV. The vaccination strategy of 20 baits per km^2 ^may be optimal, but needs some corrections in the spring period, with more attention to the latest time of baits distribution.

## Competing interests

The authors declare that they have no competing interests.

## Authors' contributions

DZ initiated, participated in the design of the study and the epidemiological investigation and performed the statistical analysis. GP participated in TTC marker investigation and carried out the immunoassays. RL and VS registered all the data, conceived of the study, participated in its design and coordination, helped to draft the manuscript, revised the manuscript. DZ, GP, RL, VS were active in writing interpretation of results and drawing the discussion. All authors read, improved the writing, approved the final and revised according to the reviewer's comments manuscript.

## Supplementary Material

Additional file 1 **Epidemiological situation of rabies in Lithuania, 2000-2005**. The table contains statistical data including total number of investigated rabies suspected samples, total number of rabies positive and percents of positive samples (domestic and wildlife animals) in Lithuania 2000 to 2005. Click here for file
