# Supplementary material for: Raccoon dog rabies surveillance and post-vaccination monitoring in Lithuania 2006 to 2010
Source: Acta Vet Scand. 2011 Nov 15;53(1):58. doi: 10.1186/1751-0147-53-58 (PMC3227601; doi:10.1186/1751-0147-53-58)
Supplement: Additional file 1 — Epidemiological situation of rabies in Lithuania, 2000-2005. The table contains statistical data including total number of investigated rabies suspected samples, total number of rabies positive and percents of positive samples (domestic and wildlife animals) in Lithuania 2000 to 2005. [file 1751-0147-53-58-S1.DOC]

Additional file 1: Table 1 Epidemiological situation of rabies in Lithuania, 2000-2005

| Year | Total No. of tested samples | Domestic animals | | | | | | Wildlife animals | | | | | | | |
| --- | --- | --- | --- | --- | --- | --- | --- | --- | --- | --- | --- | --- | --- | --- | --- |
|  |  | Dogs | Cats | Cattle | Other  domestic | Total  No. of  positive cases | % | Red foxes | Raccoon  dogs | Martens | Badgers | Other  mustilides | Other  wildlife | Total  No. of positive cases | % |
| 2000 | 1549 | 43 | 58 | 173 | 9 | 283 | 18.3 | 273 | 235 | 0 | 10 | 50 | 3 | 571 | 36.9 |
| 2001 | 1631 | 34 | 57 | 91 | 9 | 191 | 11.7 | 199 | 239 | 0 | 6 | 38 | 7 | 489 | 30.0 |
| 2002 | 1639 | 46 | 70 | 120 | 15 | 251 | 15.3 | 271 | 316 | 30 | 6 | 51 | 7 | 681 | 42.0 |
| 2003 | 1989 | 56 | 81 | 152 | 23 | 312 | 15.7 | 378 | 299 | 81 | 11 | 16 | 11 | 796 | 40.0 |
| 2004 | 2123 | 39 | 34 | 65 | 7 | 145 | 6.8 | 197 | 161 | 28 | 1 | 13 | 8 | 408 | 19.2 |
| 2005 | 2901 | 89 | 92 | 146 | 13 | 340 | 11.7 | 533 | 599 | 114 | 8 | 48 | 10 | 1312 | 45.2 |
| Total | 11832 | 307 | 392 | 747 | 76 | 1522 |  | 1851 | 1849 | 253 | 42 | 216 | 46 | 4257 |  |
| % | 100 | 2.6 | 3.3 | 6.3 | 0.6 | 12.9 |  | 15.6 | 15.6 | 2.1 | 0.4 | 1.8 | 0.5 | 36.0 |  |
| 2001-2005 Total No. of positive cases in domestic and wildlife animals 5787 (49.0% of tested samples) | | | | | | | | | | | | | | | |
